# HepG2-Based Designer Cells with Heat-Inducible Enhanced Liver Functions

**DOI:** 10.3390/cells11071194

**Published:** 2022-04-01

**Authors:** Hiroyuki Kitano, Yoshinori Kawabe, Masamichi Kamihira

**Affiliations:** Department of Chemical Engineering, Faculty of Engineering, Kyushu University, Fukuoka 819-0395, Japan; ha-ru56@kyudai.jp (H.K.); kawabe@chem-eng.kyushu-u.ac.jp (Y.K.)

**Keywords:** human hepatoma, HepG2, liver-enriched transcription factor, heat-inducible gene expression

## Abstract

Functional human hepatocytes have been a pivotal tool in pharmacological studies such as those investigating drug metabolism and hepatotoxicity. However, primary human hepatocytes are difficult to obtain in large quantities and may cause ethical problems, necessitating the development of a new cell source to replace human primary hepatocytes. We previously developed genetically modified murine hepatoma cell lines with inducible enhanced liver functions, in which eight liver-enriched transcription factor (LETF) genes were introduced into hepatoma cells as inducible transgene expression cassettes. Here, we establish a human hepatoma cell line with heat-inducible liver functions using HepG2 cells. The genetically modified hepatoma cells, designated HepG2/8F_HS, actively proliferated under normal culture conditions and, therefore, can be easily prepared in large quantities. When the expression of LETFs was induced by heat treatment at 43 °C for 30 min, cells ceased proliferation and demonstrated enhanced liver functions. Furthermore, three-dimensional spheroid cultures of HepG2/8F_HS cells showed a further increase in liver functions upon heat treatment. Comprehensive transcriptome analysis using DNA microarrays revealed that HepG2/8F_HS cells had enhanced overall expression of many liver function-related genes following heat treatment. HepG2/8F_HS cells could be useful as a new cell source for pharmacological studies and for constructing bioartificial liver systems.

## 1. Introduction

The development of functional human hepatic cells has the potential to have a great impact on the medical and pharmaceutical fields. Hepatocytes can be applied for transplantation therapy, constructing bioartificial liver devices, metabolic evaluations during drug screening, and liver disease models. However, the use of primary human hepatocytes is limited because of low availability caused by potential ethical issues and the low proliferative capacity of the cells under in vitro culture conditions. A previously investigated, the means of obtaining functional human hepatocytes is expanding cultures of primary hepatocytes [1,2,3] and then inducing hepatic differentiation from stem cells such as embryonic stem (ES) cells and induced pluripotent stem (iPS) cells [4,5,6,7,8]. However, these methods require time-consuming and costly processes to obtain large numbers of homogeneous functional hepatocytes.

Hepatoma cells have numerous advantages in terms of accessibility, ease of handling, high proliferative capacity, and phenotypic stability. Among them, human hepatoma HepG2 cells have been widely used for various hepatic studies such as drug-toxicity evaluations [9,10], constructing bioartificial liver systems [11,12], and hepatitis B virus infection models [13,14]. However, the liver function levels of hepatoma cells are significantly lower than primary hepatocytes, only exhibiting limited liver functions. Here, we have developed a new cell source for hepatocyte research by taking a synthetic biology approach to induce hepatic functions in HepG2 cells while maintaining their high proliferative potential.

Synthetic biology works to develop designer cells in which artificially programmed functions are installed [15], whereby designer cells precisely control the target gene expression from synthetic gene circuits constructed by combining characterized genetic components [16,17]. In the construction of artificial gene circuits, promoters that drive gene transcription are important components. Promoter systems that respond to chemicals [18,19], metabolites [20,21], and environmental stimuli [22,23] have been developed. We have previously developed a heat-inducible promoter system with transcriptional amplification that combines heat shock protein (HSP) promoters with a tetracycline-responsive transactivator (tTA) system [24]. tTA is a fusion protein of tetracycline repressor (tetR) and herpes simplex virus-derived transcriptional activation domain (VP16) that strongly activates a synthetic promoter composed of the tetR-responsive element (TRE) sequence and cytomegalovirus (CMV) minimum promoter (TRE/P_CMVmin_). In this system, the HSP promoter is used as a trigger for tTA expression. Although the HSP promoter induces downstream gene expression in response to heat treatment, its activity is not as strong as that of viral-derived promoters such as the CMV promoter. Therefore, tTA induced by heat treatment and then further amplified by the TRE/P_CMVmin_ promoter forms a positive-feedback loop of tTA expression. At the same time, the target gene expression under the control of the TRE/P_CMVmin_ promoter is also induced upon heat treatment and maintained thereafter.

**Figure 1:**
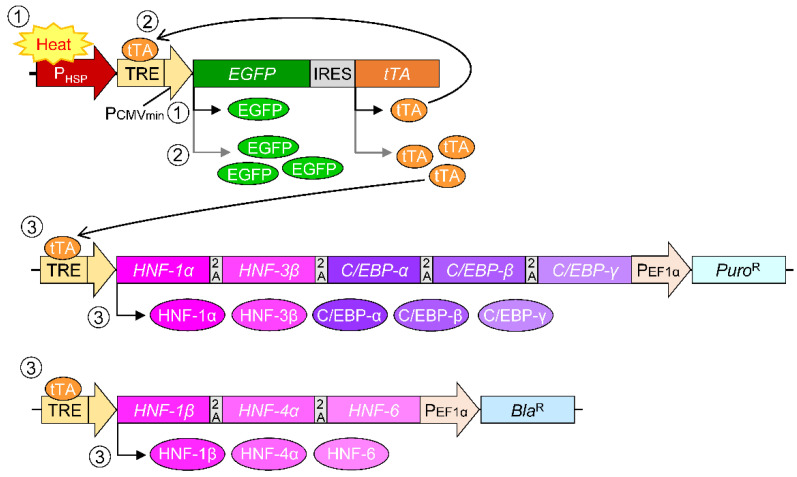
Schematic representation of the heat-inducible expression of liver-enriched transcription factor (LETF) genes mediated by artificial transactivator (tTA) with a positive feedback loop of tTA expression. (1) The heat shock protein 70B’ (HSP70B’) promoter is activated by heat treatment and induces the expression of enhanced green fluorescent protein (EGFP) and tetracycline-responsive artificial transactivator (tTA). (2) tTA binds to the tetracycline repressor protein-response element sequence (TRE) to activate cytomegalovirus minimum promoter (P_CMVmin_), which induces further tTA expression, establishing a transcriptional positive feedback loop. (3) The amplified tTA also promotes the expression of liver-enriched transcription factor (LETF) genes under the control of the TRE/P_CMVmin_ promoter. LETF genes linked by a 2A peptide sequence are co-expressed by a single promoter. To select the transduced cells, the two vectors included a puromycin resistance (*Puro^R^*) gene and a blasticidin resistance (*Bla^R^*) gene under the control of the elongation factor 1 alpha (EF1α) promoter. IRES, internal ribosomal entry site; HNF, hepatocyte nuclear factor; C/EBP, CCAAT/enhancer binding protein.

We have previously established a mouse hepatoma cell line with inducible enhanced liver functions, in which eight liver-enriched transcription factor (LETF) genes were transduced into murine hepatoma Hepa1-6 cells as the tetracycline-inducible expression units [25,26,27]. The genetically modified hepatoma cells maintained a hepatocyte phenotype through transcriptional regulation of liver-related genes by overexpression of LETF genes. In this study, we have established a human hepatoma cell line with inducible enhanced liver functions by introducing eight human-derived LETF genes under control of the TRE/P_CMVmin_ promoter into the previously developed human hepatoma cell line HepG2-HSP [28]. The heat-inducible synthetic promoter system described above was introduced into HepG2 cells, and tTA and enhanced green fluorescent protein (EGFP) were expressed via positive feedback of tTA transcription in response to heat treatment [28]. Thus, we established HepG2/8F_HS cells, in which eight LETF genes are overexpressed upon heat treatment (Figure 1). HepG2/8F_HS cells actively proliferated under normal culture conditions, and enhanced liver functions were induced by heat treatment via overexpression of LETF genes. Three-dimensional tissue-like spheroid culture was performed using HepG2/8F_HS cells, which further enhanced liver functions. Finally, overall gene expression profiles in HepG2/8F_HS cultures with and without heat treatment were analyzed using DNA microarrays. HepG2/8F_HS cells could be useful as a new cell source for pharmacological studies and constructing bioartificial liver systems.

## 2. Materials and Methods

### 2.1. Cell Culture

HepG2 and engineered HepG2 cells were cultured in high-glucose Dulbecco’s modified Eagle’s medium (DMEM) (Sigma-Aldrich, St. Louis, MO, USA) supplemented with 10% fetal bovine serum (FBS) (Biowest, Nuaillé, France), antibiotics (100 U/mL potassium penicillin G and 0.1 mg/mL streptomycin sulfate) (Fujifilm Wako Pure Chemical, Osaka, Japan), and 3.7 mg/mL NaHCO_3_ (Fujifilm Wako Pure Chemical). Cells were cultured at 37 °C in a 5% (*v*/*v*) CO_2_ incubator.

For monolayer culture, cells were seeded into collagen-coated 24-well plates (AGC Techno Glass, Shizuoka, Japan) at a density of 4.0 × 10^4^ cells/well. The next day (day 0), cells were subjected to heat treatment. The medium was changed every other day, and the harvested medium was applied for liver function analyses.

To fractionate EGFP-positive cells, cells were seeded into collagen-coated 100-mm dishes (AGC Techno Glass) and exposed to heat treatment when the cell density reached 80% confluency. The next day (day 0), the cell population with high EGFP fluorescence intensity was sorted using an SH800 cell sorter (Sony, Tokyo, Japan). Cells were cultured at 1.0 × 10^5^ cells/well in collagen-coated 24-well plates for monolayer culture, or at 2.3 × 10^4^ or 4.7 × 10^4^ cells/well (50 or 100 cells/microwell) in EZSPHERE SP 24-well plates (AGC Techno Glass) for spheroid culture. The culture medium was replaced with fresh medium every other day. To replace the entire culture medium for spheroid cultures, the spheroids were collected by centrifugation and reseeded into ultra-low adhesion plates (Corning, New York, NY, USA). Harvested medium was used for analysis.

### 2.2. Transposon Vector Plasmids

The PiggyBac-based transposon vector plasmids PB513B-1/TRE-hHNF1α-hHNF3β-hCEBPα-hCEBPβ-hCEBPγ-EF1α-Puro and PB513B-1/TRE-hHNF1β-hHNF4α-hHNF6-EF1α-Bla [29], which encode the expression units of eight LETF genes under control of the TRE/P_CMVmin_ promoter, were used to introduce the LETF genes into HepG2-HSP cells. In these vectors, each LETF gene is linked through a 2A peptide sequence, and LETF proteins are produced from single transcripts.

### 2.3. Transfection and Establishment of Stable Cell Clones

HepG2-HSP cells were cultured at 4.0 × 10^5^ cells/well on collagen-coated 6-well plates (AGC Techno Glass). The next day, the transposon vector plasmids PB513B-1/TRE-hHNF1α-hHNF3β-hCEBPα-hCEBPβ-hCEBPγ-EF1α-Puro and PB513B-1/TRE-hHNF1β-hHNF4α-hHNF6-EF1α-Bla and the PiggyBac transposase expression vector plasmid (System Biosciences, Palo Alto, CA, USA) were transfected into cells using Lipofectamine 3000 (Thermo Fisher Scientific, Waltham, MA, USA) in accordance with the manufacturer’s instructions. After 6 h incubation, the medium was changed with fresh medium. To obtain stably transduced bulk cells, cells were cultured in the presence of 10 µg/mL puromycin (Thermo Fisher Scientific) and 5 µg/mL blasticidin (Thermo Fisher Scientific) for 12 d from day 2 post-transfection. Cell clones were established by the limiting dilution method.

### 2.4. Determining Heat Treatment Conditions

The day after seeding HepG2-HSP cells, the culture plates were sealed and submerged into a water bath at 41 °C, 42 °C, 43 °C, or 44 °C for 0.5, 1, or 2 h. After heat treatment, the cells were cultured at 37 °C in a 5% (*v*/*v*) CO_2_ incubator. On day 5 of culture, the number of viable cells and the percentage of EGFP-positive cells were measured. The number of viable cells was counted by the trypan blue dye exclusion method, and EGFP-positive cells were measured using an SH800 cell sorter.

### 2.5. Immunostaining

On day 3, cells were fixed using 4% paraformaldehyde (Nara Pathology Laboratory, Nara, Japan) and permeabilized using phosphate-buffered saline (PBS) containing 0.1% Triton X-100. After blocking using 1% bovine serum albumin (Fujifilm Wako Pure Chemical) in PBS, cells were incubated with goat anti-albumin antibody (A80-129A; Bethyl, Montgomery, TX, USA) and then with rhodamine-conjugated rabbit anti-goat IgG-R (sc-3945; Santa Cruz Biotechnology, Dallas, TX, USA); nuclei were stained with 4′,6-diamidino-2-phenylindole (DAPI) (Sigma-Aldrich). Stained cells were observed using a BZ-X810 fluorescence microscope (Keyence, Osaka, Japan).

### 2.6. Liver Function Analyses

The concentration of albumin produced in the medium was measured by enzyme-linked immunosorbent assay (ELISA) using a Human Albumin ELISA Quantitation Set (E80-129; Bethyl). To measure the ammonia removal rate, the culture supernatant was collected after 24 h incubation in fresh medium (500 µL/well) containing 2 mM NH_4_Cl (Fujifilm Wako Pure Chemical). The ammonia concentration was measured using a commercially available kit (Ammonia-Test Wako; Fujifilm Wako Pure Chemical). The removal rate was determined by calculating the amount of ammonia reduction. To measure cytochrome P450 activity, the culture supernatant was collected after 1 h incubation in fresh medium (300 μL/well) with 3.0 μM luciferin substrate (Luciferin-IPA; Promega, Madison, WI, USA). The luciferin produced by CYP3A4 enzymatic activity in hepatic cells was quantified using luciferase (Luciferin Detection Reagent; Promega), and the luminescence intensity was measured using a Glo-max Luminometer (Promega). These assays were performed in accordance with the manufacturer’s instructions.

### 2.7. Gene Expression Analysis Using DNA Microarrays

On day 5, the cells were collected and frozen as cell pellets. Total RNA was extracted using TRIzol (Thermo Fisher Scientific) and purified using the RNeasy Mini Kit (Qiagen, Hilden, Germany). Gene expression analysis was performed using a SurePrint G3 Human Gene Expression v3 8 × 60 K Microarray Kit (Agilent Technologies, Santa Clara, CA, USA). The quality of total RNA was checked using an Agilent 2200 TapeStation (Agilent Technologies). Each 50 ng of total RNA was labeled using an Agilent Low-Input QuickAmp Labeling Kit, One-Color, in accordance with the manufacturer’s protocols. Data were normalized using the quantile method. Microarray analysis was performed by Cell Innovator (Fukuoka, Japan). Microarray data are available in the ArrayExpress database (www.ebi.ac.uk/arrayexpress) under accession number E-MTAB-11486 (accessible from 31 May 2022).

### 2.8. Statistical Analysis

The Student’s *t*-test was used to compare all data, with *p* < 0.05 considered significant differences.

## 3. Results

### 3.1. Optimization of Heat Treatment Conditions

To determine the optimal heat treatment conditions to induce the tTA-positive feedback loop, HepG2-HSP cells were heated at 41 °C, 42 °C, 43 °C, or 44 °C for 0.5, 1, or 2 h. For each condition, the number of viable cells (Figure S1a) and the percentage of EGFP-positive cells (Figure S1b) were measured 5 d after heat treatment. EGFP expression was induced by heat treatment under all conditions in which cells were alive. More than 90% of cells were EGFP-positive following heat treatment at 42 °C for 2 h, 43 °C for 1 and 2 h, and 44 °C for 0.5 h, while most cells died following heat treatment at 44 °C for 1 or 2 h. Among the conditions showing a high percentage of EGFP-positive cells, a 30-min treatment at 43 °C was determined to be the optimal condition because it showed minimal effects on cell proliferation after treatment.

### 3.2. Screening HepG2/8F_HS Cells and Liver Function Analyses

After transfecting HepG2-HSP cells with PB513B-1/TRE-hHNF1α-hHNF3β-hCEBPα-hCEBPβ-hCEBPγ-EF1α-Puro and PB513B-1/TRE-hHNF1β-hHNF4α-hHNF6-EF1α-Bla, transgenic bulk cells were obtained by double-drug (puromycin and blasticidin) screening. To evaluate the liver functions induced by LETF overexpression, HepG2 and transgenic bulk cells were cultured with or without heat treatment. The number of viable cells, albumin secretion rate, ammonia removal rate, and cytochrome P450 activity were measured over the culture period (Figure S2). The parental HepG2 cells, with or without heat treatment, exhibited very little of the three functions. In contrast, the transgenic bulk cells with heat treatment showed increased albumin secretion rate and cytochrome P450 activity of 18- and 5-fold, respectively, compared with those of HepG2 cells, while the ammonia removal rate was not improved.

Next, cell clones were obtained from the bulk cells by the limiting dilution method, and then cultured for 5 d with or without heat treatment. Subsequently, most clones showed cell growth arrest and enhanced albumin secretion (Figure S3a,b). Clone #29, which showed enhanced albumin secretion, was designated as HepG2/8F_HS cells, and used in subsequent experiments.

**Figure 2:**
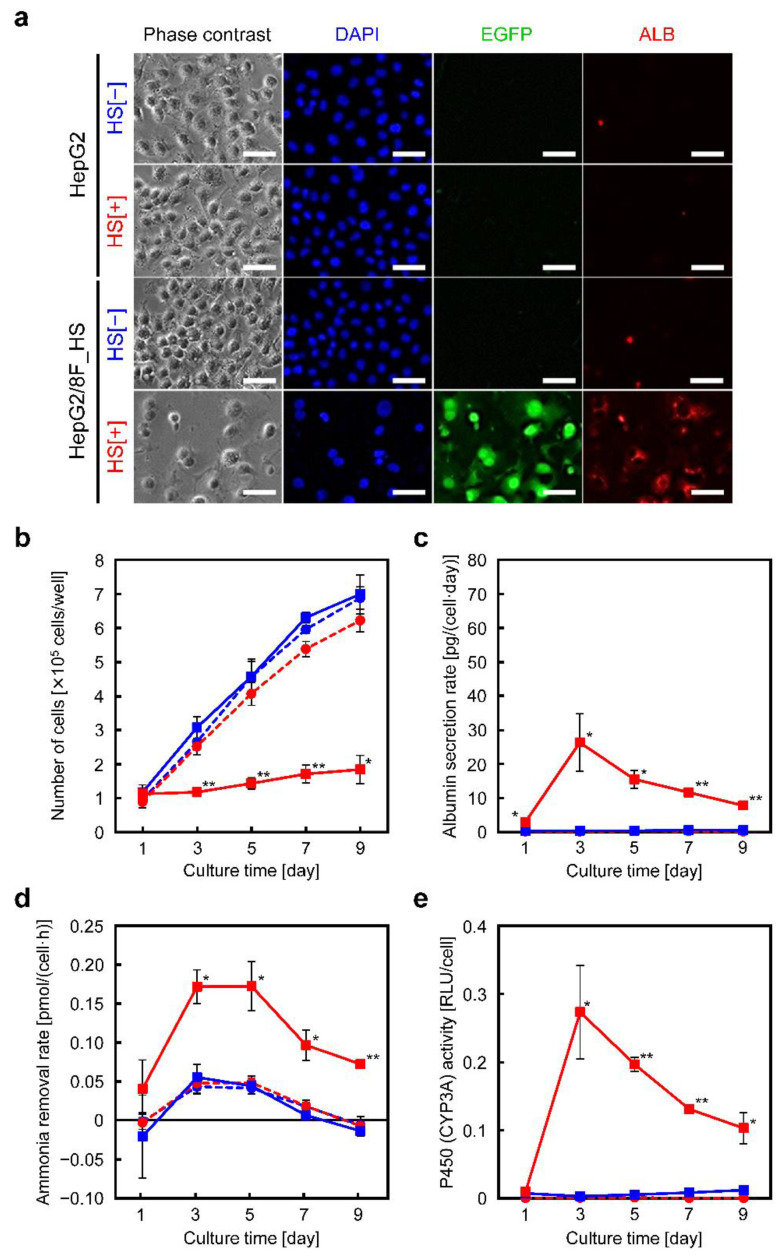
Morphological observations and liver function analyses of HepG2 and HepG2/8F_HS cells in monolayer culture: (**a**) Phase contrast and fluorescence images of HepG2 and HepG2/8F_HS cells with (HS[+]) or without (HS[−]) heat treatment. DAPI, nuclei; EGFP, enhanced green fluorescent protein; ALB, albumin. Scale bars = 50 µm. (**b**–**e**) Liver function analyses during the culture of HepG2 (circles) and HepG2/8F_HS (squares) cells. The number of cells (**b**), albumin secretion rate (**c**), ammonia removal rate (**d**), and cytochrome P450 (CYP3A4) activity (**e**) were measured. HS[−], blue symbols; HS[+], red symbols. Data are presented as means ± standard deviations (*n* = 3); * *p* < 0.05, ** *p* < 0.01 vs. HS[−].

We observed the cell morphology of HepG2 and HepG2/8F_HS cells after 3 d of culture with and without heat treatment. No differences in cell morphology or proliferation rates were found between HepG2 cells with and without heat treatment. In contrast, heat-treated HepG2/8F_HS cells showed suppressed proliferation, increased cell size, and binuclear cells were observed, as is seen in cultured primary hepatocytes (Figure 2a) [30]. Additionally, EGFP fluorescence was observed in HepG2/8F_HS cells after heat treatment, indicating induced expression of the transduced LETF genes (Figure 2a). Overexpression of LETF genes in heat-treated HepG2/8F_HS cells was also confirmed by western blot analysis (Figure S4). Furthermore, albumin expression was observed by immunocytochemistry, which indicated liver function. In contrast, no or faint albumin expression was observed in HepG2 cells and HepG2/8F_HS cells without heat treatment, while strong fluorescence (indicating albumin expression) was observed in HepG2/8F_HS cells with heat treatment (Figure 2a). The number of viable cells, albumin secretion rate, ammonia removal rate, and cytochrome P450 activity were measured over the culture period (Figure 2b–e). In HepG2/8F_HS cells, cell proliferation was inhibited by heat treatment, but the cells proliferated slightly after day 9. The albumin secretion rate, ammonia removal rate, and cytochrome P450 activity were significantly enhanced in heat-treated HepG2/8F_HS cells. The induced liver functions peaked on day 3 and gradually decreased thereafter, but remained higher than those of parental HepG2 cells on day 9. We also measured the ammonia removal rate of other clones and found that all clones with enhanced albumin production exhibited ammonia removal capacity (Figure S3c).

### 3.3. Fractioning Cells with Enhanced Liver Functions on the Basis of EGFP Fluorescence Intensity

**Figure 3:**
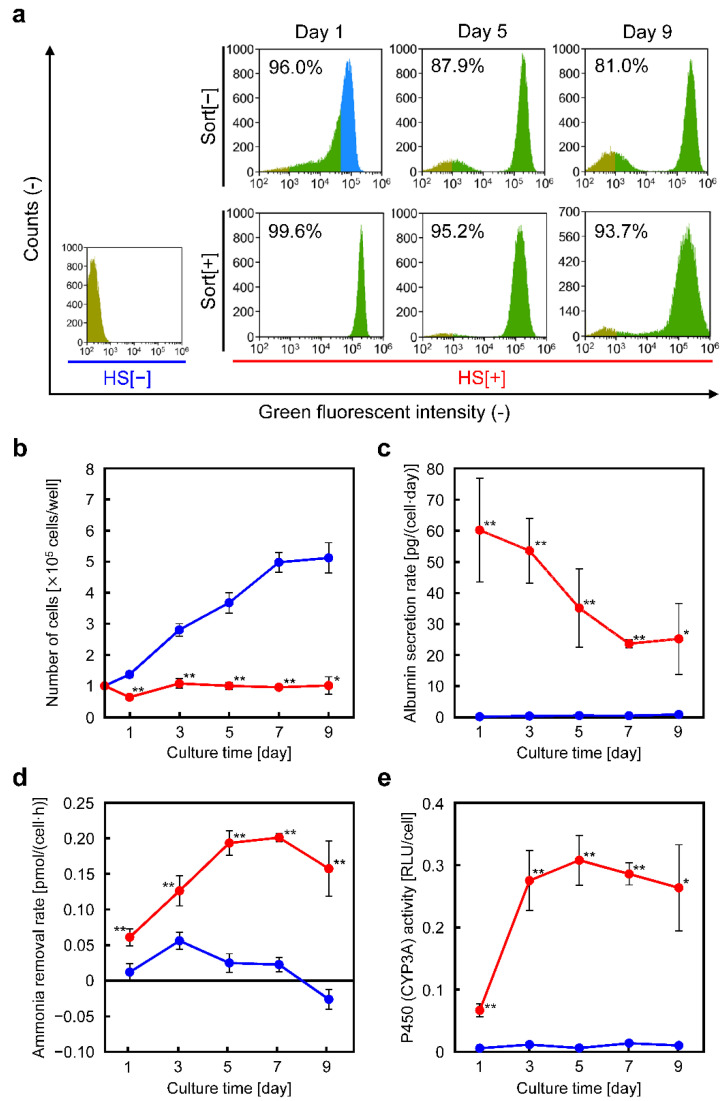
EGFP-based cell sorting and liver function analyses of heat-treated HepG2/8F_HS cells in monolayer culture: (**a**) EGFP flowcytometry of heat-treated HepG2/8F_HS cells with (Sort[+]) or without (Sort[−]) sorting during culture. EGFP-negative (yellow area) and positive (blue/green area) cell fractions are shown. The percentages of EGFP-positive cells are indicated in the histograms. The EGFP-positive fraction (blue area) was collected by cell sorting. (**b**–**e**) Liver function analyses of the EGFP-positive fraction of heat-treated HepG2/8F_HS cells. The number of cells (**b**), albumin secretion rate (**c**), ammonia removal rate (**d**), and cytochrome P450 (CYP3A4) activity (**e**) were measured. HS[−], blue symbols; HS[+], red symbols. Data are presented as means ± standard deviations (*n* = 3); * *p* < 0.05, ** *p* < 0.01 vs. HS[−].

The percentage of EGFP-positive cells after heat-treating HepG2/8F_HS cells was measured over time (Figure 3a). On day 1 after heat treatment, 96% of the cells showed EGFP fluorescence. On day 5, the cells were divided into two populations: one with higher EGFP fluorescence intensity and the other with lower EGFP fluorescence intensity. On day 9, the percentage of cells with low EGFP fluorescence increased, while the population with high EGFP fluorescence maintained its fluorescence intensity. In addition, a population of EGFP-negative cells with high cell density was observed locally on the culture plate (Figure S5). On the day after heat treatment, cells with high EGFP fluorescence intensity (fraction of the blue region in Figure 3a) were collected, and the percentage of EGFP-positive cells, the number of viable cells, and liver functions were measured for the cells over time. The results showed that the percentage of EGFP-positive cells remained higher than that of the bulk cells on day 9 (Figure 3a). The cells with high EGFP fluorescence intensity did not proliferate even after 9 d of culture (Figure 3b and Figure S5). The peak albumin secretion rate was increased 2.3-fold compared with that of the bulk cells, and was maintained the same level as the peak without sorting, even on day 9 (Figure 3c). Although the ammonia removal rate and cytochrome P450 activity were not significantly improved compared with those of the bulk cells, the duration of the high levels was extended (Figure 3d,e). Thus, fractioning EGFP-positive cells after heat treatment was effective for maintaining the induced enhanced liver function over a long period of time.

### 3.4. Spheroid Culture of HepG2/8F_HS Cells

**Figure 4:**
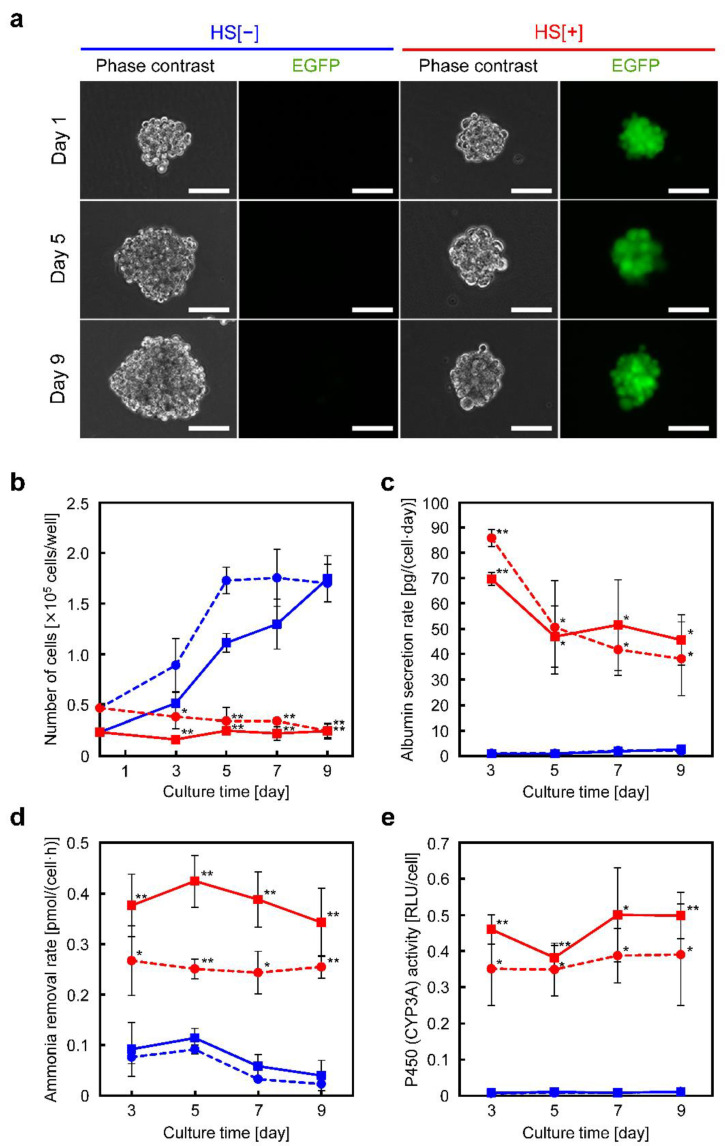
Morphological observations and liver function analyses of HepG2/8F_HS cells in spheroid culture: (**a**) Phase contrast and fluorescence images of HepG2/8F_HS spheroids with (HS[+]) or without (HS[−]) heat treatment. Cells were seeded at 50 cells/microwell and observed during culture. EGFP, enhanced green fluorescent protein. Scale bars = 100 µm. (**b**–**e**) Liver function analyses during the spheroid culture of HepG2/8F_HS cells. The number of cells (**b**), albumin secretion rate (**c**), ammonia removal rate (**d**), and cytochrome P450 (CYP3A4) activity (**e**), were measured. HS[−], blue symbols; HS[+], red symbols. Cells were seeded at 50 cells/microwell (squares) or 100 cells/microwell (circles). Data are presented as means ± standard deviations (*n* = 3); * *p* < 0.05, ** *p* < 0.01 vs. HS[−].

To evaluate the ability to induce further enhanced liver function, HepG2/8F_HS cells were cultured as spheroids. When cells were seeded onto a non-adherent culture substrate capable of inducing spheroid formation, spherical cell aggregates with a diameter of approximately 100 µm were formed the day after beginning culture (Figure 4a). In spheroids formed with HepG2/8F_HS cells without heat treatment, the cells gradually proliferated and reached a size of approximately 200 µm in diameter on day 9. In contrast, when the spheroids were formed from heat-treated cells (with fractionation of EGFP-positive cells), the cells did not proliferate, and the diameter of the spheroids remained at approximately 100 µm on day 9. The number of viable cells, albumin secretion rate, ammonia removal rate, and cytochrome P450 activity were measured over the culture period. As was the case for monolayer cultures, cells proliferated without heat treatment, while cell proliferation was suppressed with heat treatment (Figure 4b). Under the condition of 100 cells/microwell, dead cells were observed in the late stages of culture. The albumin secretion rate was slightly enhanced compared with monolayer culture, and the induced function was maintained at a high level (Figure 4c). The ammonia removal rate and cytochrome P450 activity (Figure 4d,e) were higher at 50 cells/microwell than at 100 cells/microwell, and the values were 2.1- and 1.6-fold higher, respectively, compared with those at the peak of monolayer culture (Figure 3d,e).

### 3.5. Whole-Genome Expression Analysis of HepG2/8F_HS Cells Using DNA Microarrays

**Figure 5:**
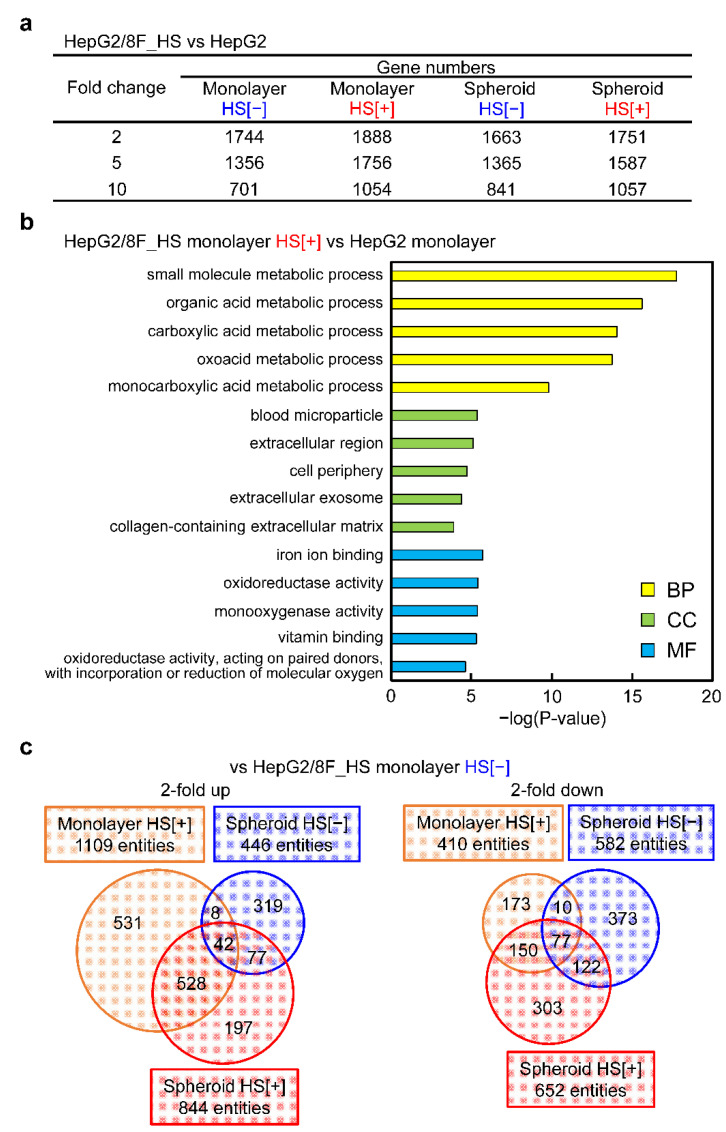
Whole-genome expression analysis of HepG2 and HepG2/8F_HS cells using DNA microarrays: (**a**) Relative fold change in genes expressed in HepG2/8F_HS cells compared with parental HepG2 cells. The total number of genes that showed fold increase or decrease in both monolayer and spheroid cultures with (HS[+]) or without (HS[−]) heat treatment. (**b**) Gene ontology (GO) analysis of 1888 genes that increased more than 2-fold in HepG2/8F_HS monolayer HS[+] compared with HepG2 monolayer. The five most significantly (*p* < 0.05) enriched GO terms in the biological process (BP), cellular component (CC), and molecular function (MF) categories are presented. (**c**) Venn diagrams showing the number of genes with more than 2-fold increase (left) or decrease (right) in cultures of HepaG2/8F_HS cells as monolayer HS[+], and spheroid HS[−] and HS[+],compared with monolayer HS[−].

Comprehensive gene expression analysis was performed using DNA microarrays. Compared with parental HepG2 cells, the expression of many genes in HepG2/8F_HS cells was altered in all conditions. In particular, in monolayer culture with heat-treated HepG2/8F_HS cells, the expression of 1888, 1756, and 1054 genes was changed more than 2-, 5-, and 10-fold, respectively (Figure 5a). Gene ontology (GO) enrichment analysis was performed on the 1888 genes whose expression was increased more than 2-fold in heat-treated HepG2/8F_HS cells compared with parental HepG2 cells (Figure 5b). Gene ontology is a terminology system that describes the function of gene products using a common vocabulary independent of species and disciplines, and the defined terms are classified into three categories: biological processes (BP), cellular components (CC), and molecular functions (MF). The top five GO terms in the BP category that were significantly enriched were all related to metabolic processes. Next, we summarized the number of genes whose expression varied more than 2-fold in each condition relative to the monolayer culture of HepG2/8F_HS cells without heat treatment (Figure 5c). There were 570 genes shared between the monolayer culture of heat-treated cells and the spheroid culture of heat-treated cells.

**Figure 6:**
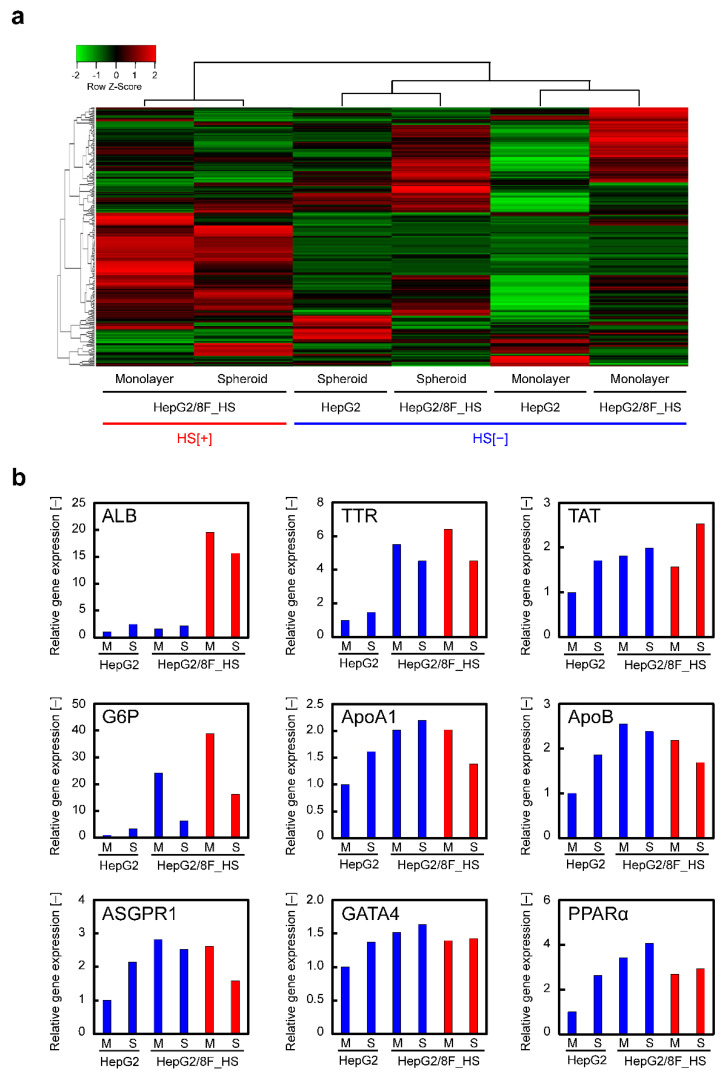
Analysis of the expression of liver-related genes in HepG2 and HepG2/8F_HS cells using DNA microarrays: (**a**) Heatmap analysis of liver-related gene expression in HepG2 and HepG2/8F_HS cells with (HS[+]) or without (HS[−]) heat treatment in monolayer and spheroid cultures. Hierarchical clustering was analyzed using the Pearson’s correlation distance/average linkage method. (**b**) Relative gene expression of liver-related genes. Cells were cultured with (red columns) or without (blue columns) heat treatment. M, monolayer; S, spheroid.

A heat map of gene expression was created for genes selected with at least 4-fold higher mRNA levels in liver compared with any other tissues (obtained from The Human Protein ATLAS) (Figure 6a and Figure S6). The introduction of LETF genes increased the expression of many liver-related genes even without heat treatment (Figure 6b). This may be due to leaky expression of the LETF genes. Importantly, a drastic increase in the expression of a large number of liver-related genes was observed in heat-treated HepG2/8F_HS cells.

## 4. Discussion

In this study, we established a human hepatoma cell line, HepG2/8F_HS, which proliferated actively under normal culture conditions and can be induced to have high liver function by thermal stimulation. Candidate cell sources for use in bioartificial livers and drug screening systems must have efficient cell proliferation capacity and stable qualities. It has been reported that approaches such as co-culture with non-parenchymal liver cells (Kupffer cells, hepatic stellate cells, hepatic sinusoidal endothelial cells, etc.) [31] and aggregation into spheroids [32] improved the function and viability of primary human hepatocytes. However, it remains difficult to expand and maintain functional primary hepatocytes for a long time. In recent years, several groups have reported the reprogramming of human primary hepatocytes into proliferative liver progenitor-like cells [1,2,3]. However, these methods need the addition of small molecules, supplements, and growth factors, as well as long culture periods to obtain highly functional hepatic cells because they go through hepatic progenitor cells. These methods also require cumbersome and costly operations due to sequential changes in culture conditions. Additionally, these cells from primary culture cause variations in liver function depending on the cell harvest lot. An alternative possible cell source is iPS cell-derived hepatocytes [4,5,6,7,8]. Although an efficient method for producing large numbers of liver buds from human iPS cells using microwell plates has been developed [33], the use of iPS cell-derived hepatocytes still involves time and high costs for cell differentiation, and heterogeneity in quality remains an issue. In contrast, HepG2/8F_HS cells use hepatoma HepG2 as parental cells, and can grow in a simple basal medium that does not require any special additives, allowing large numbers of cells to be obtained easily and rapidly. Additionally, a single heat treatment was sufficient to induce liver functions, and high liver functions were observed 3 d after heat treatment. Furthermore, preparative isolation of cells based on EGFP expression has the advantage of stabilizing the quality and maintaining the induced liver functions for a certain period of time (Figure 3). In our previous murine hepatoma cell line, in which high liver functions were also induced by thermal stimulation, we were unable to isolate functionalized cells, and hence we added mitomycin C to completely arrest cell proliferation and maintain high liver functions [27]. In contrast, functionalized HepG2/8F_HS cells can be easily separated using EGFP expression as an indicator.

In our previous study of murine hepatoma cells with inducible high liver function, there was a negative correlation between cell proliferation and high liver function induced by overexpression of LETF [25]. In this study, cell proliferation was not affected by heat treatment at 43 °C for 30 min in non-LETF-transduced cells (HepG2-HSP) (Figure S1); however, in LETF-transduced cells (HepG2/8F_HS), cell proliferation was suppressed in response to the heat-induced overexpression of LETF genes (Figure 2b). These results indicate that appropriate heat treatment could minimize the cell damage caused by heat treatment, and that overexpression of LETF genes could change the cell phenotype to show enhanced liver functions. Additionally, the expression of LETF genes in HepG2/8F_HS cells is regulated by a positive feedback loop of tTA that is triggered by heat treatment, and their expression can be monitored by EGFP fluorescence (Figure 1 and Figure 3a). On the day after heat treatment, the cells with low EGFP fluorescence intensity gradually lost their expression over subsequent passages because the tTA positive feedback loop was not working sufficiently. Cells with low EGFP fluorescence intensity or not expressing EGFP at all proliferated due to insufficient LETF gene expression. In contrast, cells with high EGFP fluorescence intensity increased their fluorescence intensity over further passages due to accumulation of EGFP by the tTA positive feedback loop. These cells showed enhanced liver functions owing to the overexpression of LETF genes and stopped proliferating. Thus, if there was a mixture of cells in which induction was not working sufficiently after heat treatment, the percentage of cells exhibiting decreased liver function that were insufficiently induced would proliferate. This resulted in a decrease in heat-induced liver functions on day 9 of culture (Figure 2c–e). When cells with high EGFP fluorescence intensity were isolated, the induced liver function was maintained (Figure 3c–e).

Many researchers have reported HepG2 spheroids to enhance hepatic function. However, the function level was very low compared with that of primary hepatocytes [32]. The heat-induced liver functions of HepG2/8F_HS cells were enhanced by forming spheroids, which could be sustained for 9 d (Figure 4). The function level was the highest ever reported for HepG2 spheroids. However, heat treatment after the formation of spheroids failed to induce sufficient liver functions. The same phenomenon was observed in previously developed drug-inducible murine hepatoma cells, where the addition of the inducer drug after spheroid formation failed to induce sufficient liver function. Thus, cell–cell interactions may affect the induction of hepatic functions via LETF overexpression in hepatoma cells.

In the liver, LETFs are highly expressed and form a transcriptional network with each other to regulate the expression of various genes related to liver functions and create the hepatocyte phenotype [34]. LETF genes have also been used for direct reprogramming of fibroblasts to hepatocytes [35,36,37]. In this study, we introduced eight LETF genes into human hepatoma HepG2 cells to overexpress them, which also enhanced the expression of various liver-related genes (Figure 6). In addition, the expression of many enzymes involved in phase I, II, and III reactions of drug metabolism and urea synthesis were also enhanced (Figure S7). Furthermore, GO enrichment analysis was performed on genes that showed more than 2-fold increased expression in heat-treated HepG2/8F_HS cells and primary hepatocytes compared with parental HepG2 cells. From this analysis, we found that 10 of the top 15 significantly enriched GO terms were common (Figure 5b and Figure S8). These results suggest that overexpressing multiple LETF genes changed the phenotype of the cells, transforming them from HepG2 cells to functional hepatocytes. Gupta et al. compared hepatic cells by RNA-seq-based transcriptome analysis and showed that HepG2 is significantly different from primary hepatocytes [38]. Although the expression levels of liver-related genes in heat-treated HepG2/8F_HS cells were significantly improved compared with parental HepG2 cells, the gene expression profile was different from that of primary hepatocytes (Figure S9). Since the same medium (DMEM+10% FBS) as usual HepG2 culture was used as the culture medium, further improvements can be expected by examining the medium components after inducing liver function.

In conclusion, we established a human hepatoma cell line, HepG2/8F_HS, in which high liver function can be induced by overexpressing eight LETF genes. Liver functions such as albumin secretion, ammonia removal, and cytochrome P450 activity were rapidly improved by heat stimulation. Additionally, using EGFP expression to sort cells with high liver function, we were able to obtain a cell population with relatively uniform expression. Furthermore, comprehensive transcriptome analysis showed increased expression of many genes related to liver functions. Therefore, HepG2/8F_HS cells are a promising new cell source for constructing bioartificial livers and chemical compound screening during drug discovery.

## Data Availability

Microarray data are available in the ArrayExpress database (https://www.ebi.ac.uk/arrayexpress) at EMBL-EBI under accession number E-MTAB-11486. The other raw data are available from the corresponding author upon request.

## Supplementary Materials

The following supporting information can be downloaded at: https://www.mdpi.com/article/10.3390/cells11071194/s1, Figure S1: Effect of heat treatment on cell growth and EGFP expression in HepG2-HSP cells. Cells were cultured at 37 °C for 5 d after heat treatment at 41 °C, 42 °C, 43 °C, or 44 °C for 0.5, 1, or 2 h. No heat treatment was set as the control. The number of viable cells (**a**) and percentage of EGFP-positive cells (**b**) were measured. Data are presented as means ± standard deviations (*n* = 3); * *p* < 0.05, ** *p* < 0.01 vs. control. C, control; EGFP, enhanced green fluorescent protein; N.D., not detected. Figure S2: Liver function analyses of HepG2 and HepG2/8F_HS bulk cells in monolayer culture. The number of cells (**a**), albumin secretion rate (**b**), ammonia removal rate (**c**), and cytochrome P450 (CYP3A4) activity (**d**) were measured during the culture of HepG2 (circles) and HepG2/8F_HS bulk (squares) cells with (red symbols) or without (blue symbols) heat treatment. Data are presented as means ± standard deviations (*n* = 3); * *p* < 0.05, ** *p* < 0.01 vs. without heat treatment. Figure S3: Liver function analyses of HepG2/8F_HS clones in monolayer culture. The number of cells (**a**), albumin secretion rate (**b**), and ammonia removal rate (**c**) were measured with (red columns) or without (blue columns) heat treatment on day 5. Figure S4: Western blot analysis of eight liver-enriched transcription factors in HepG2/8F_HS cells. Cells were seeded into collagen-coated 100-mm dishes and exposed to heat treatment at 43 °C for 30 min when the cell density reached 80% confluency. Three days after heat treatment, cells were detached from the dish using trypsin and collected by centrifugation. The cells were suspended in PBS containing 1 mM PMSF (P7626; Sigma-Aldrich) and disrupted by sonication. Cell lysate samples were applied for Wes automated capillary western blotting (Model No. 004-600A-N001; ProteinSimple, San Jose, CA, USA). Primary antibodies were purchased from Santa Cruz Biotechnology (anti-HNF-1α, sc-393668; anti-HNF-1β, sc-130407; anti-HNF-3β, sc-101060; anti-HNF-4α, sc-374229; anti-HNF-6, sc-376167; anti-C/EBP-α, sc-166258; anti-C/EBP-β, sc-7962; and anti-C/EBP-γ, sc-517003) or Cell Signaling Technology (anti-GAPDH, #2118). Anti-mouse secondary antibody was purchased from ProteinSimple (042-205). Figure S5: Microscopy images showing EGFP expression by heat-treated HepG2/8F_HS cells. Cells were cultured for 9 d after heat treatment with (Sort[+]) or without (Sort[−]) sorting. Scale bars = 100 µm. Figure S6: Heatmap analysis of liver-related gene expression in HepG2 and HepG2/8F_HS cells with (HS[+]) or without (HS[−]) heat treatment in monolayer and spheroid cultures. Gene names used for analysis in Figure 6a are listed in the right column. Figure S7: Relative gene expression of liver-related genes in HepG2 and HepG2/8F_HS cells. (**a**) Drug metabolism response-related genes. (**b**) Urea cycle enzyme genes. Cells were cultured with (red columns) or without (blue columns) heat treatment. M, monolayer; S, spheroid. Figure S8: Gene ontology (GO) analysis of genes with more than 2-fold increase in adult primary human hepatocytes (HC5-25) compared with HepG2. Microarray data of HC5-25 cells (GSM3963162) were obtained from the Gene Expression Omnibus of the National Center for Biotechnology Information. The five most significantly (*p* < 0.05) enriched GO terms in the biological process (BP), cellular component (CC), and molecular function (MF) categories are presented. Figure S9: Heatmap analysis of liver-related gene expression of HepG2 and heat-treated HepG2/8F_HS cells in comparison with primary human hepatocytes. DNA microarray data for 187271 (GSM3963168), JFC (GSM3927233) and HC5-25 (GSM3963162) cells were obtained from the Gene Expression Omnibus of the National Center for Biotechnology Information. Hierarchical clustering was analyzed using the Pearson’s correlation distance/average linkage method.
