# Supplementary material for: HepG2-Based Designer Cells with Heat-Inducible Enhanced Liver Functions
Source: Cells. 2022 Apr 1;11(7):1194. doi: 10.3390/cells11071194 (PMC8997820; doi:10.3390/cells11071194)
Supplement: Supplementary file 1 [file cells-11-01194-s001.zip › cells-1648438-supplementary.pdf]

Supplementary Figures

HepG2-Based Designer Cells with Heat-Inducible Enhanced Liver Functions

Hiroyuki Kitano, Yoshinori Kawabe and Masamichi Kamihira *

Figure captions

Figure S1. Effect of heat treatment on cell growth and EGFP expression in HepG2-HSP cells. Cells were cultured at 37°C for 5 d after heat treatment at 41°C, 42°C, 43°C, or 44°C for 0.5, 1, or 2 h. No heat treatment was set as the control. The number of viable cells (**a**) and percentage of EGFP-positive cells (**b**) were measured. Data are presented as means ± standard deviations ($n = 3$); $^*P < 0.05$, $^{**}P < 0.01$ vs. control. C, control; EGFP, enhanced green fluorescent protein; N.D., not detected.

Figure S2. Liver function analyses of HepG2 and HepG2/8F_HS bulk cells in monolayer culture. The number of cells (**a**), albumin secretion rate (**b**), ammonia removal rate (**c**), and cytochrome P450 (CYP3A4) activity (**d**) were measured during the culture of HepG2 (circles) and HepG2/8F_HS bulk (squares) cells with (red symbols) or without (blue symbols) heat treatment. Data are presented as means ± standard deviations ($n = 3$); $^*P < 0.05$, $^{**}P < 0.01$ vs. without heat treatment.

Figure S3. Liver function analyses of HepG2/8F_HS clones in monolayer culture. The number of cells (**a**), albumin secretion rate (**b**), and ammonia removal rate (**c**) were measured with (red columns) or without (blue columns) heat treatment on day 5.

Figure S4. Western blot analysis of eight liver-enriched transcription factors in HepG2/8F_HS cells. Cells were seeded into collagen-coated 100-mm dishes and exposed to heat treatment at 43°C for 30 min when the cell density reached 80% confluency. Three days after heat treatment, cells were detached from the dish using trypsin and collected by centrifugation. The cells were suspended in PBS containing 1 mM PMSF (P7626; Sigma-Aldrich) and disrupted by sonication. Cell lysate samples were applied for Wes automated capillary western blotting (Model No. 004-600A-N001; ProteinSimple, San Jose, CA, USA). Primary antibodies were purchased from Santa Cruz Biotechnology (anti-HNF-1α, sc-393668; anti-HNF-1β, sc-130407; anti-HNF-3β, sc-101060; anti-HNF-4α, sc-374227; anti-HNF-6, sc-376167; anti-C/EBP-α, sc-166258; anti-C/EBP-β, sc-7962; and anti-C/EBP-γ, sc-517003) or Cell Signaling Technology (anti-GAPDH, #2118). Anti-mouse secondary antibody was purchased from ProteinSimple (042-205).

Figure S5. Microscopy images showing EGFP expression by heat-treated HepG2/8F_HS cells. Cells were cultured for 9 d after heat treatment with (Sort[+]) or without (Sort[−]) sorting. Scale bars = 100 μm.

Figure S6. Heatmap analysis of liver-related gene expression in HepG2 and HepG2/8F_HS cells with (HS[+]) or without (HS[−]) heat treatment in monolayer and spheroid cultures. Gene names used for analysis in Figure 6a are listed in the right column.

Figure S7. Relative gene expression of liver-related genes in HepG2 and HepG2/8F_HS cells. (**a**) Drug metabolism response-related genes. (**b**) Urea cycle enzyme genes. Cells were cultured with (red columns) or without (blue columns) heat treatment. M, monolayer; S, spheroid.

Figure S8. Gene ontology (GO) analysis of genes with more than 2-fold increase in adult primary human hepatocytes (HC5-25) compared with HepG2. Microarray data of HC5-25 cells (GSM3963162) were obtained from the Gene Expression Omnibus of the National Center for Biotechnology Information. The five most significantly ($P < 0.05$) enriched GO terms in the biological process (BP), cellular component (CC), and molecular function (MF) categories are presented.

Figure S9. Heatmap analysis of liver-related gene expression of HepG2 and heat-treated HepG2/8F_HS cells in comparison with primary human hepatocytes. DNA microarray data for 187271 (GSM3963168), JFC (GSM3927233) and HC5-25 (GSM3963162) cells were obtained from the Gene Expression Omnibus of the National Center for Biotechnology Information. Hierarchical clustering was analyzed using the Pearson's correlation distance/average linkage method.

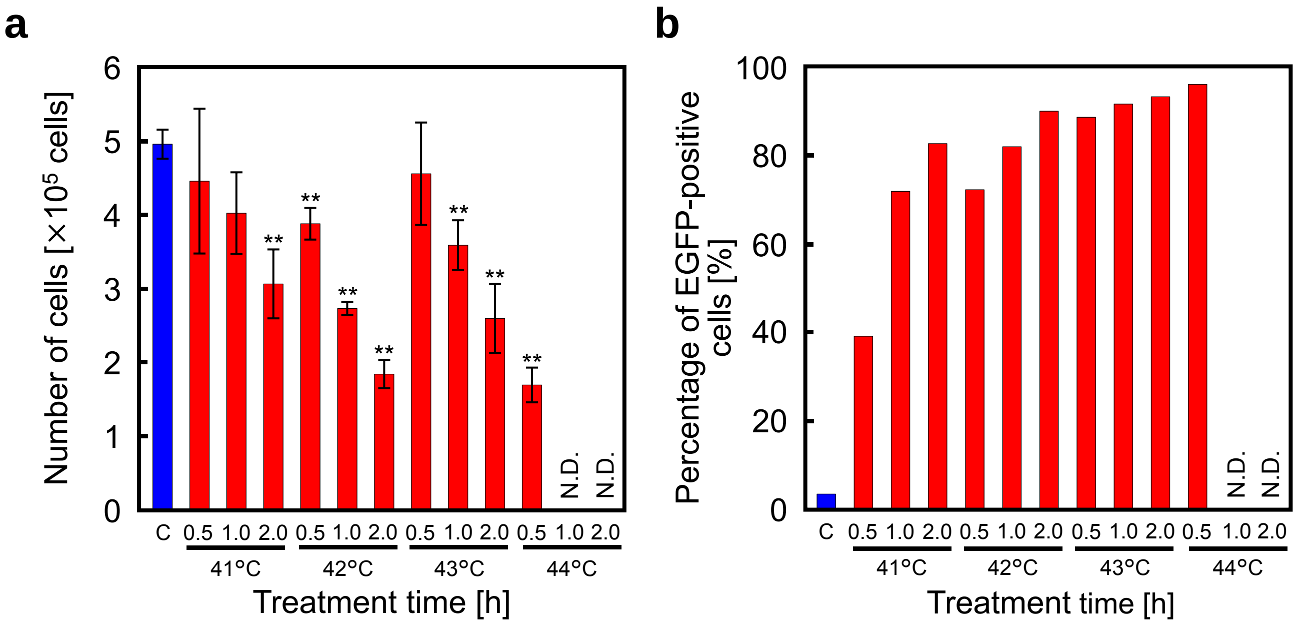

Figure S1. Kitano *et al.*

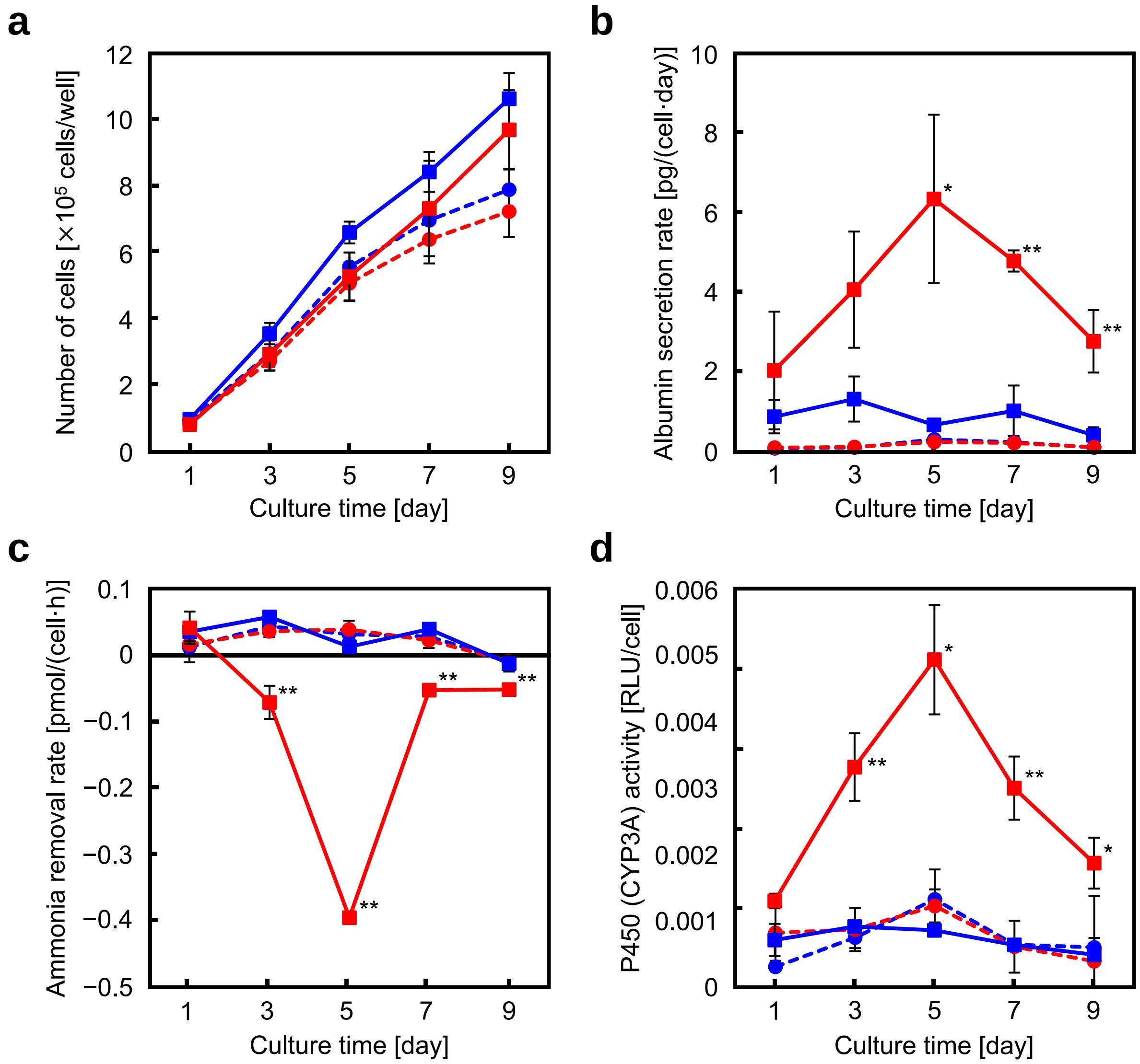

Figure S2. Kitano *et al.*

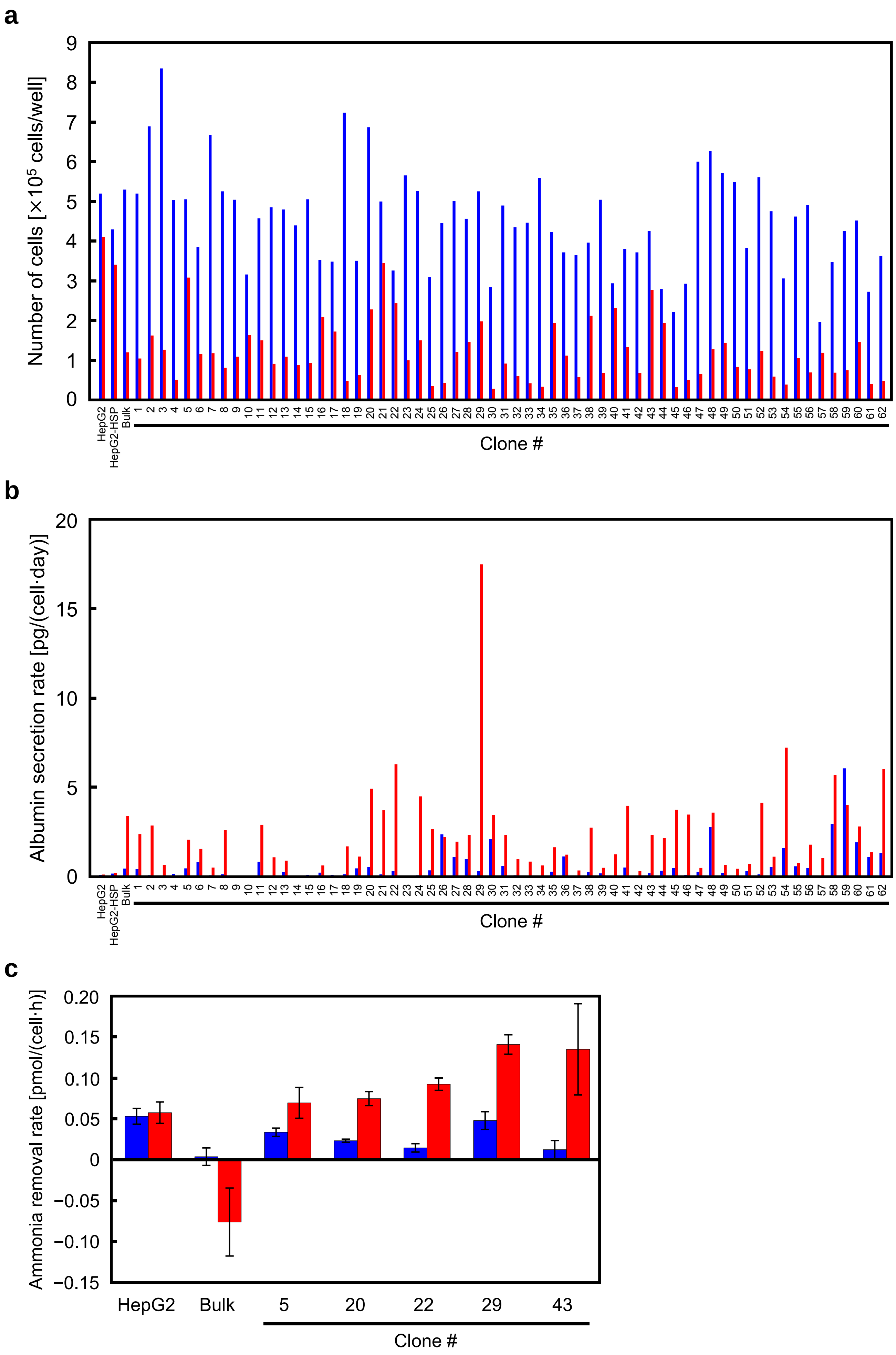

Figure S3. Kitano *et al.*

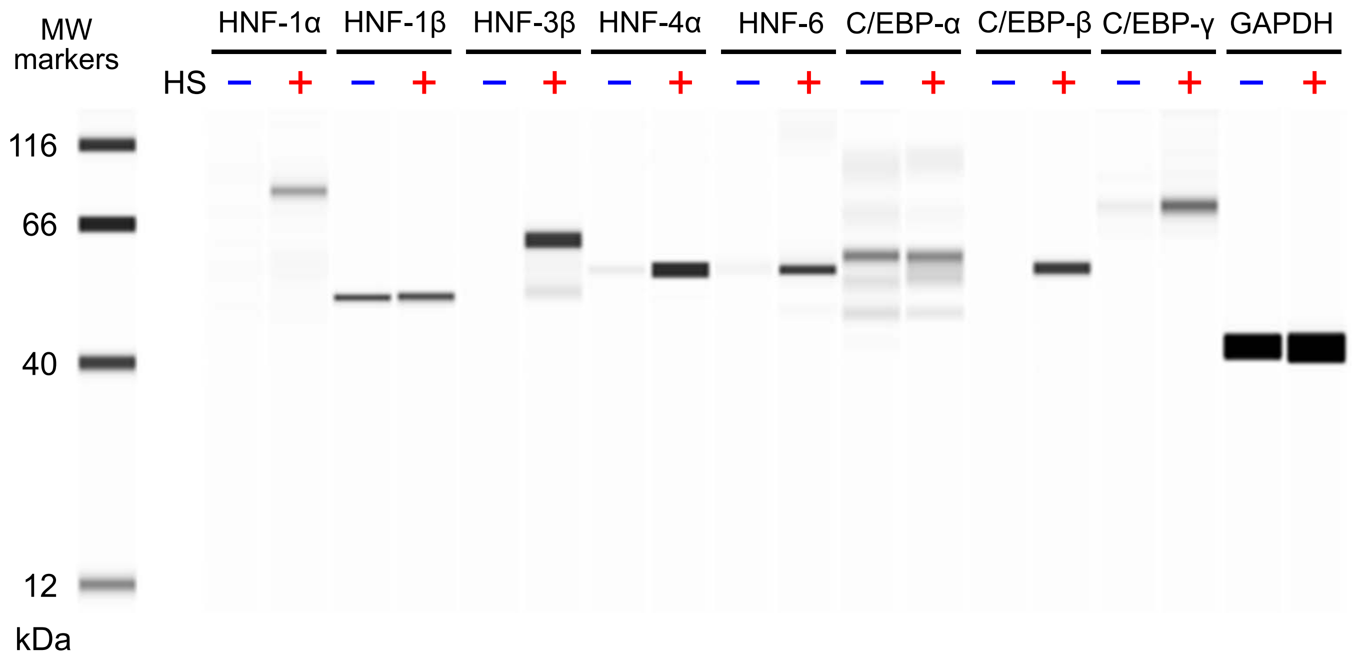

Figure S4. Kitano *et al.*

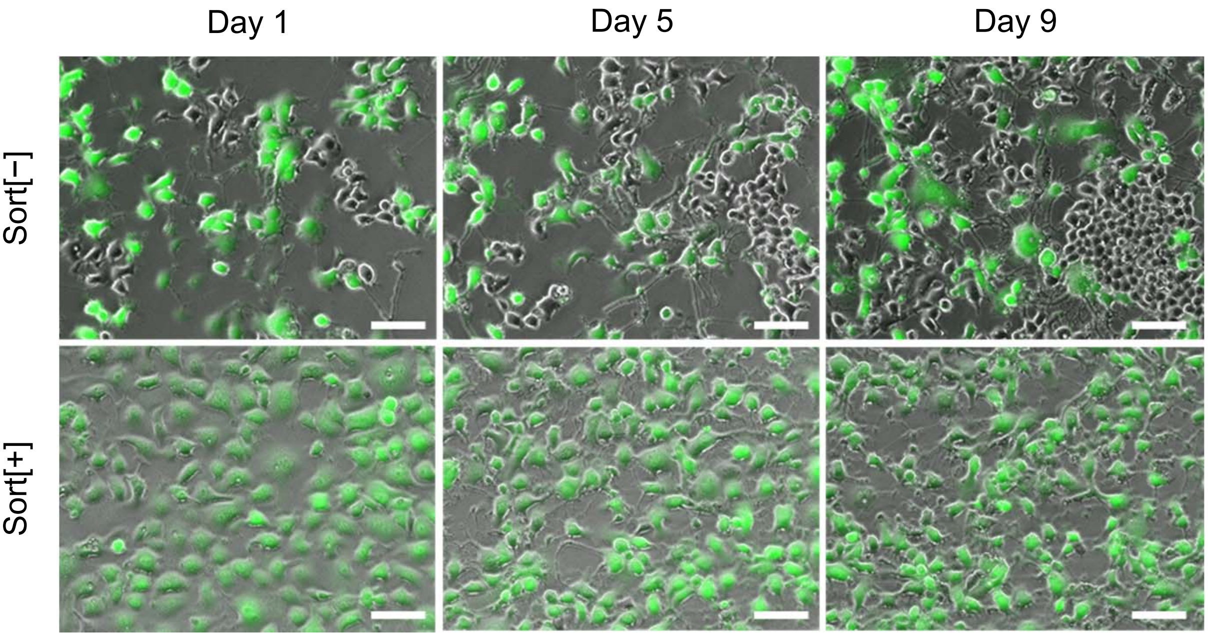

Figure S5. Kitano *et al.*

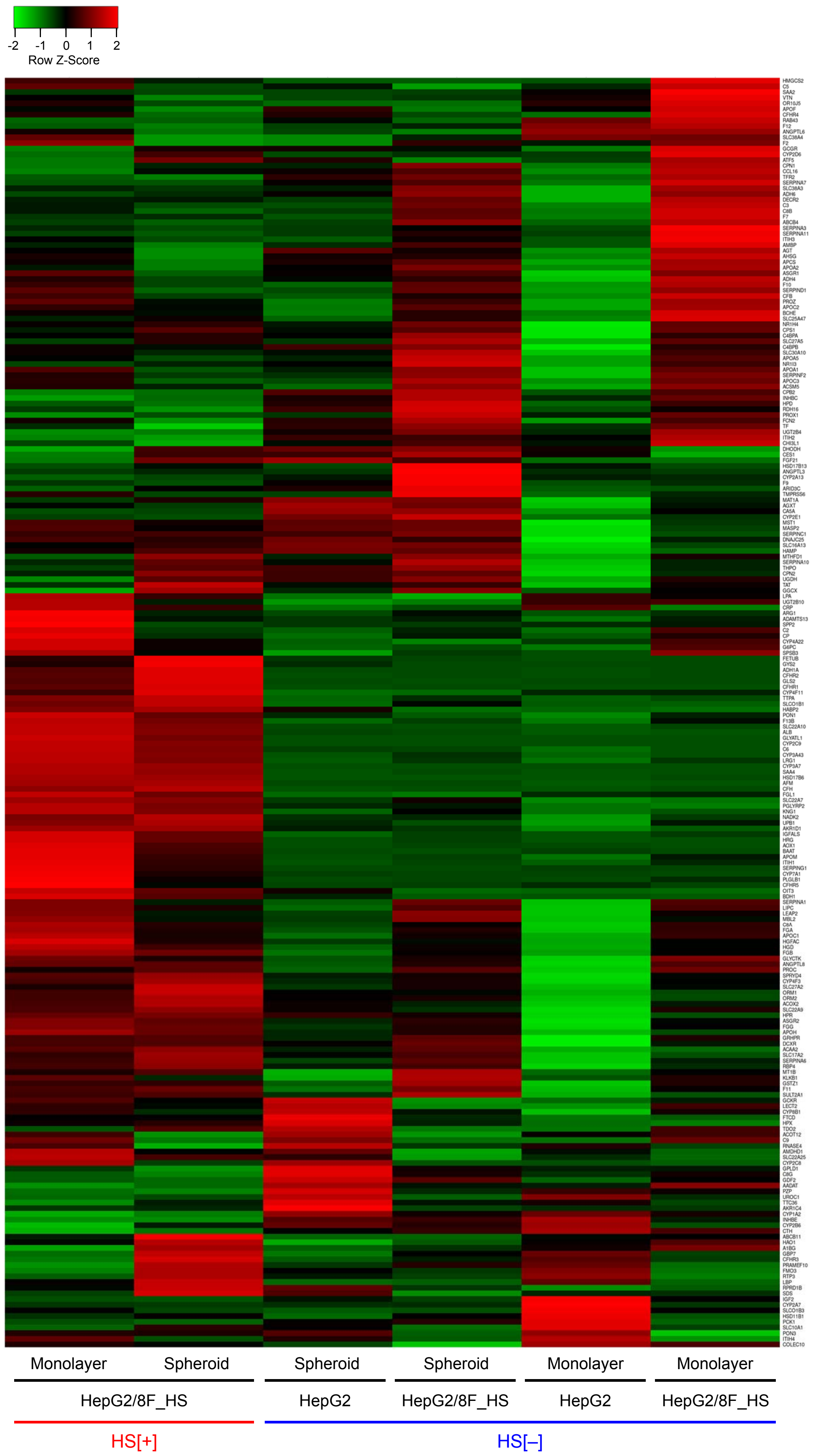

Figure S6. Kitano *et al.*

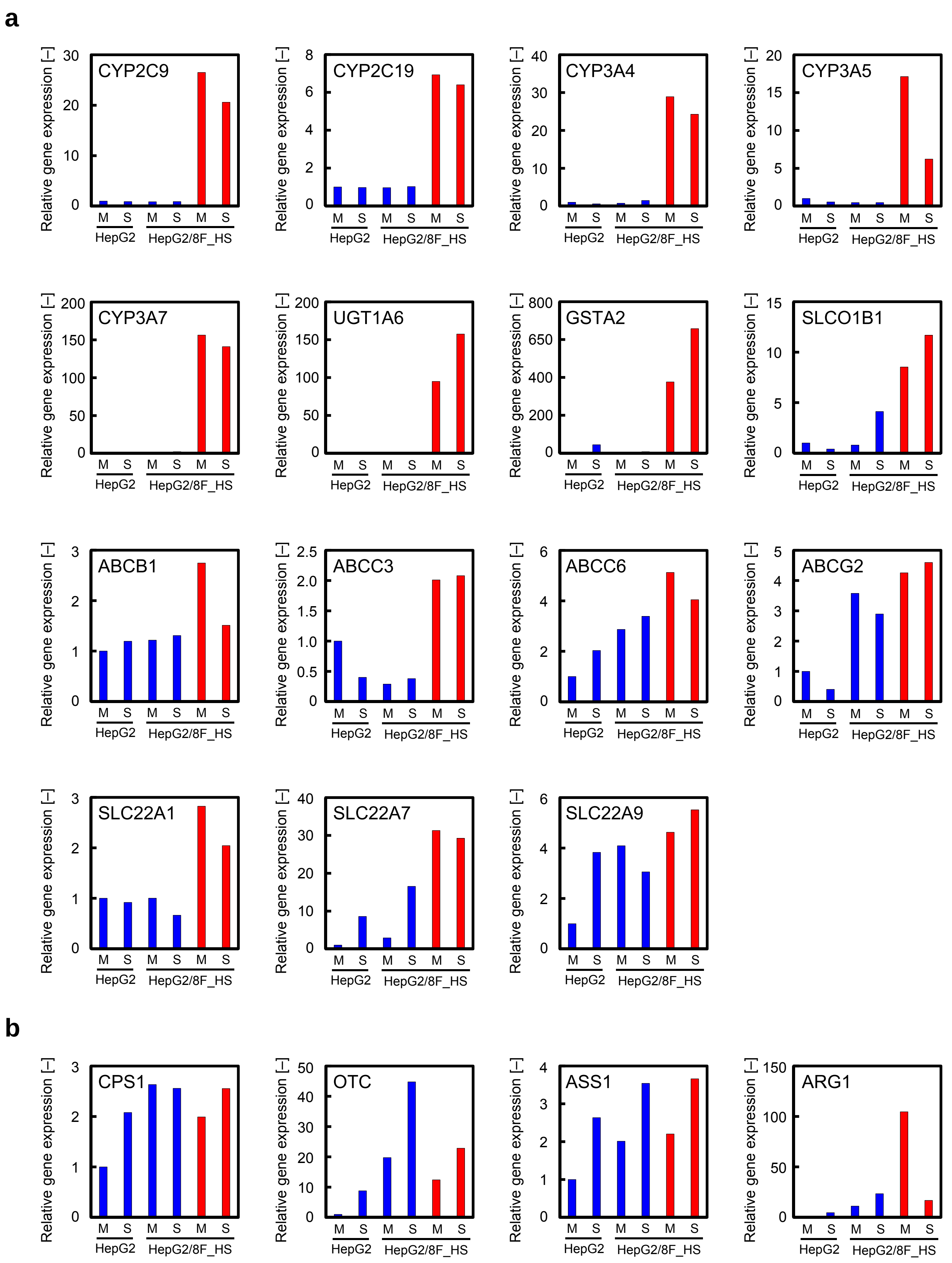

Figure S7. Kitano *et al.*

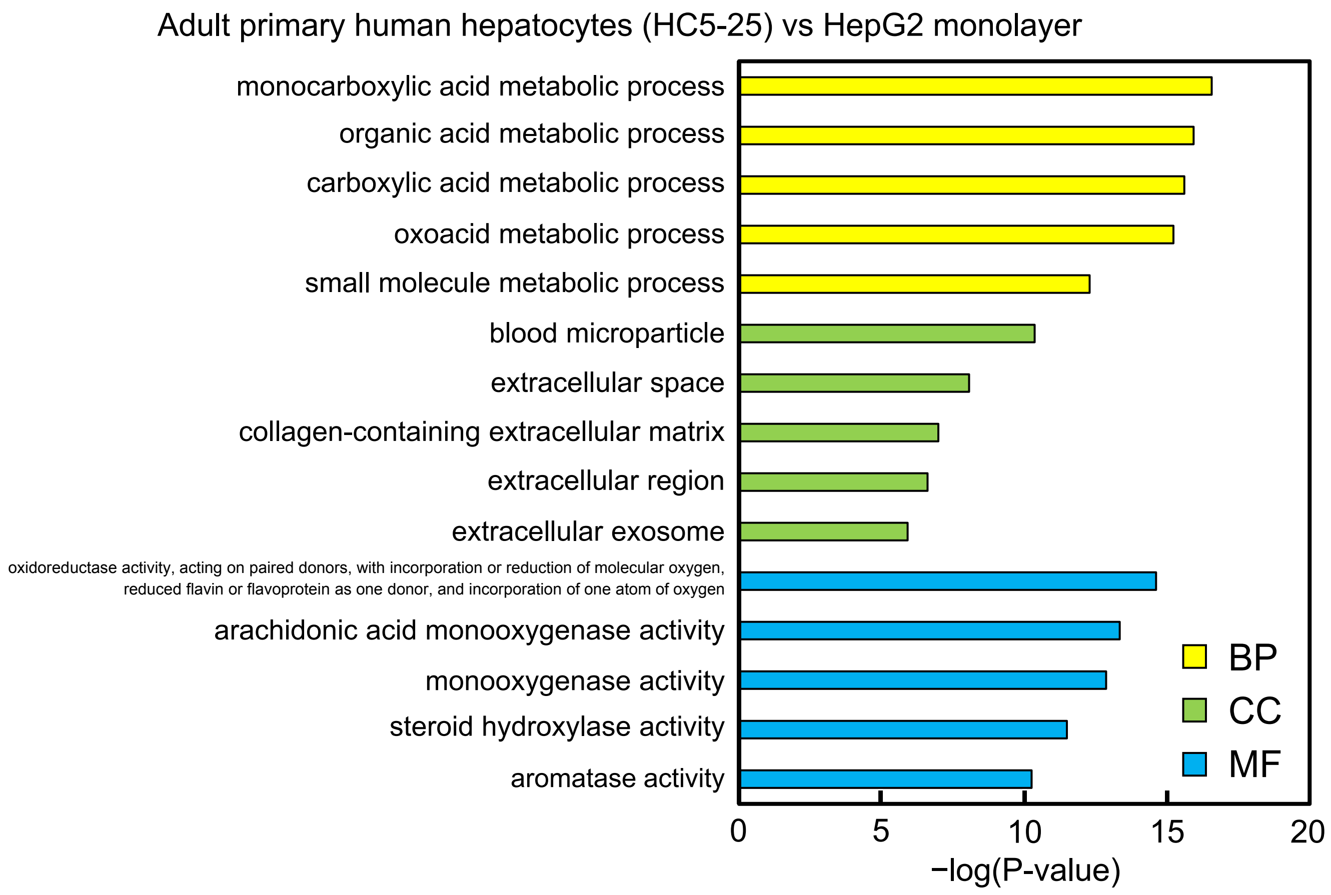

Figure S8. Kitano *et al.*

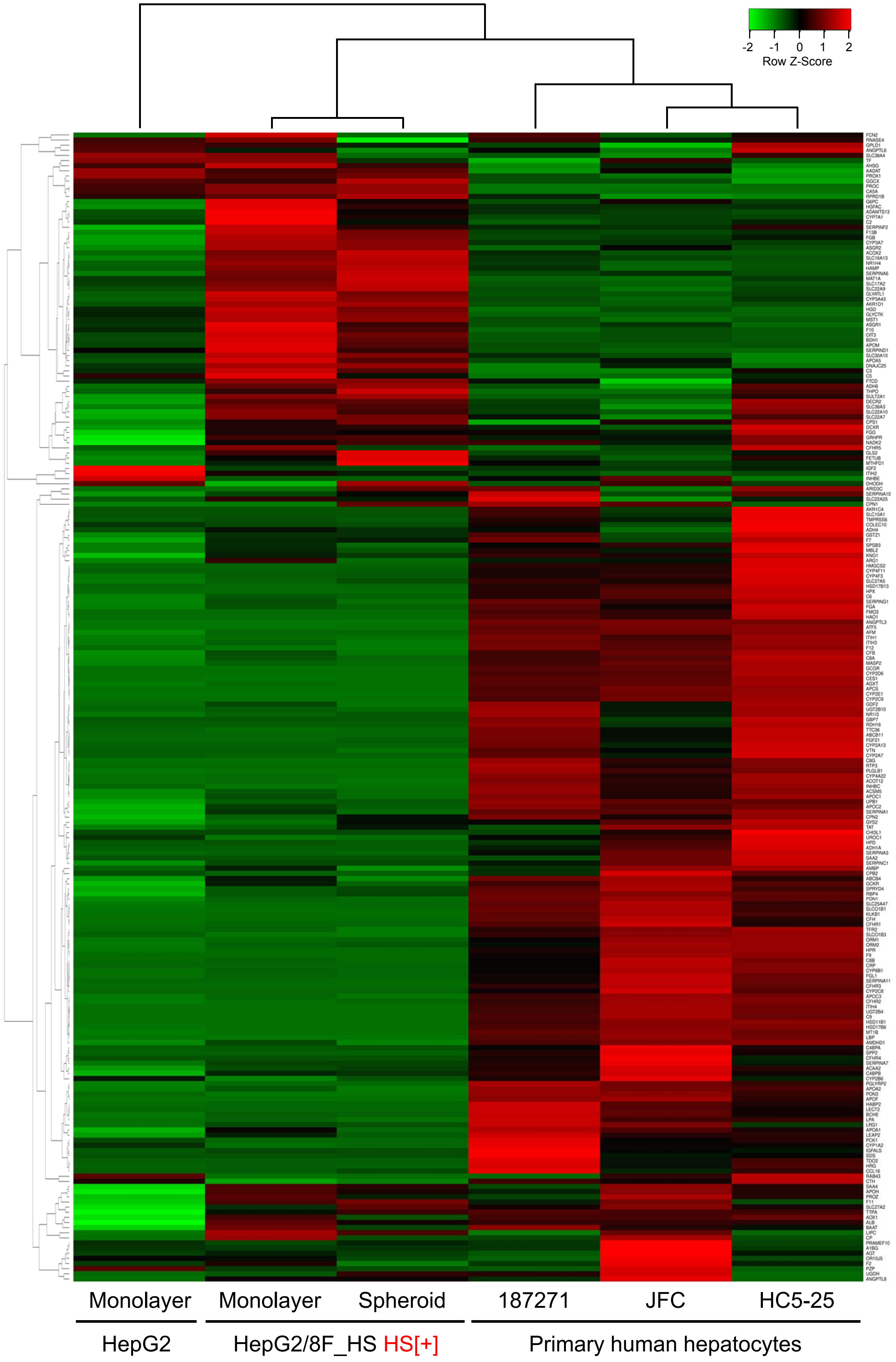

Figure S9. Kitano *et al.*
